# Leveling up evidence syntheses: filling conceptual gaps of the role of midwifery in health systems through a network analysis

**DOI:** 10.1186/s13104-022-06094-0

**Published:** 2022-06-21

**Authors:** Cristina A. Mattison, Kirsty Bourret, Michelle L. Dion

**Affiliations:** 1 Department of Women and Children’s Health, 2Karolinska Institutet, Tomtebodavägen 18A, Solna, 171 77 Sweden; 2 Department of Obstetrics and Gynecology, McMaster University, McMaster Midwifery Research Centre, 1280 Main St. West, HSC-4H26, L8S 4K1 Hamilton, Canada; 3 Department of Political Science, McMaster University, 1280 Main St. West, KTH-533, Hamilton, ON L8S 4M4 Canada

**Keywords:** Network analysis, Evidence synthesis, Mixed methods, Midwifery, Critical interpretive synthesis, Health systems, Health policy, Sexual and reproductive health and rights

## Abstract

**Objective:**

In the research note, our main objective is to explore the value of combining an evidence synthesis with a network analysis. The discussion is based on a critical interpretive synthesis, which combines systematic review methodology with qualitive inquiry, and ‘research concept’ network analysis focused on understanding the roles of midwives in health systems. The interpretative analytic approach of a critical interpretive synthesis has a high explanatory value by allowing for the review of a diverse body of literature and is well-suited to delving into areas that are not well understood, such as midwifery.

**Results:**

Network analyses use graphs to represent relationships between concepts and brought to light important additional insights into the literature that were not present in the evidence synthesis alone. Given the lack of theoretical development in the area of midwifery in health systems, the critical interpretive synthesis allowed for the generation of concepts used to inform a theoretical framework, while the novel application of an exploratory network analysis deepened understanding of conceptual areas of saturation within the field, as well as identifying critical gaps in the literature.

## Introduction

The use of evidence review methodologies to synthesize quantitative or qualitative research has grown substantially in the past decade. Deciding on an approach to evidence synthesis, ranging from traditional systematic reviews to integrated approaches is key. Yet there is limited guidance available on choosing the knowledge synthesis method most appropriate to answer a particular research question [1, 2].

As we continue to advance approaches to knowledge synthesis, clarity is needed to find the right methodological match, while innovating to maximize insights. In this note, we demonstrate how a network analysis complements an evidence synthesis by mapping the connections between concepts instead of simply summarizing them. We use a case of a critical interpretive synthesis (CIS) combined with a ‘research concept’ network analysis to understand the roles of midwives in health systems. To address the lack of theoretical understanding of midwives’ roles, a CIS was used to generate theory, while the novel addition of an exploratory network analysis yielded important insights into conceptual areas of over saturation and gaps.

CIS is a relatively new review approach and has been increasingly used to synthesize quantitative or qualitative evidence [3, 4]. The approach’s flexibility, emphasis on theory generation, and suitability for framework development are its key advantages [3]. CIS is an inductive approach to literature analysis, using conventional systematic review processes while incorporating qualitative inquiries to examine both empirical and non-empirical literature [5]. Criticisms of the methodology centre on lack of transparency in the application of the review [2, 6]. Additionally, CIS and evidence review methodologies generally, describe concepts in the literature without explicitly analysing connections between concepts.

Network analyses are a methodology for understanding relationships through graphs. The network itself is represented by a structure that consists of a group of nodes and edges. The nodes represent the object of study and the edges represent the relationships between the objects that connect them [7]. Common applications of network analyses are found in social network analysis (e.g., understanding collaboration networks) or bibliometric network analysis (e.g. examining the relationships between publications) [8, 9].

## Main text

In this study we focused on the health profession of midwifery, as midwives are a vital contributor to improving the world’s sexual and reproductive health and rights (SRHR), but the profession has not reached its full potential [10]. While there is ample research evidence demonstrating that midwives deliver high-quality SRHR services and save lives, there is lack of understanding of the role and scope of midwifery across all levels of health systems [11–17]. We used a CIS to develop a theoretical framework, and the compass question that oriented our research asked: “Across health systems, what are the factors that influence the roles of midwives within the health system?” Details of the literature selection, screening, review by independent coders, and discrepancy resolution are explained in [18]. This paper presents a theoretical framework, which maps the key elements that influence midwives’ roles in a particular political and health system. The factors highlight the range of variables influencing the level of integration of the profession, and the cumulative effects of the barriers that lead to health systems where the profession is disempowered and marginalized [18].

### Extending the CIS by adding a network analysis

Here, we extend the CIS methodology by adding an exploratory network analysis to understand how the relevant literature on the factors that influence the roles of midwives within the health system are conceptually related. In this case, network analysis was used as a way to analyze relationships among the key health system components that emerged in the theoretical framework [19–21].

All the included records (*n* = 117) were used in the exploratory network analysis, and the nodes in this study are theoretical concepts, while edges are the interactions or relationships between the concepts [7, 19, 20]. Centrality measures are key as they help us understand what are the most central nodes in the network, and in this case, what concepts related to the role of midwives in health systems are the most central in the literature [7, 19, 20, 22]. There are a number of ways to measure centrality in a network and for the purposes of this analysis we used betweenness centrality. This type of centrality measures the importance of a node to the shortest paths through the network, and is one of the most common measures of centrality because it captures how important a node is to the flow of information across the network [7].

**Fig. 1:**
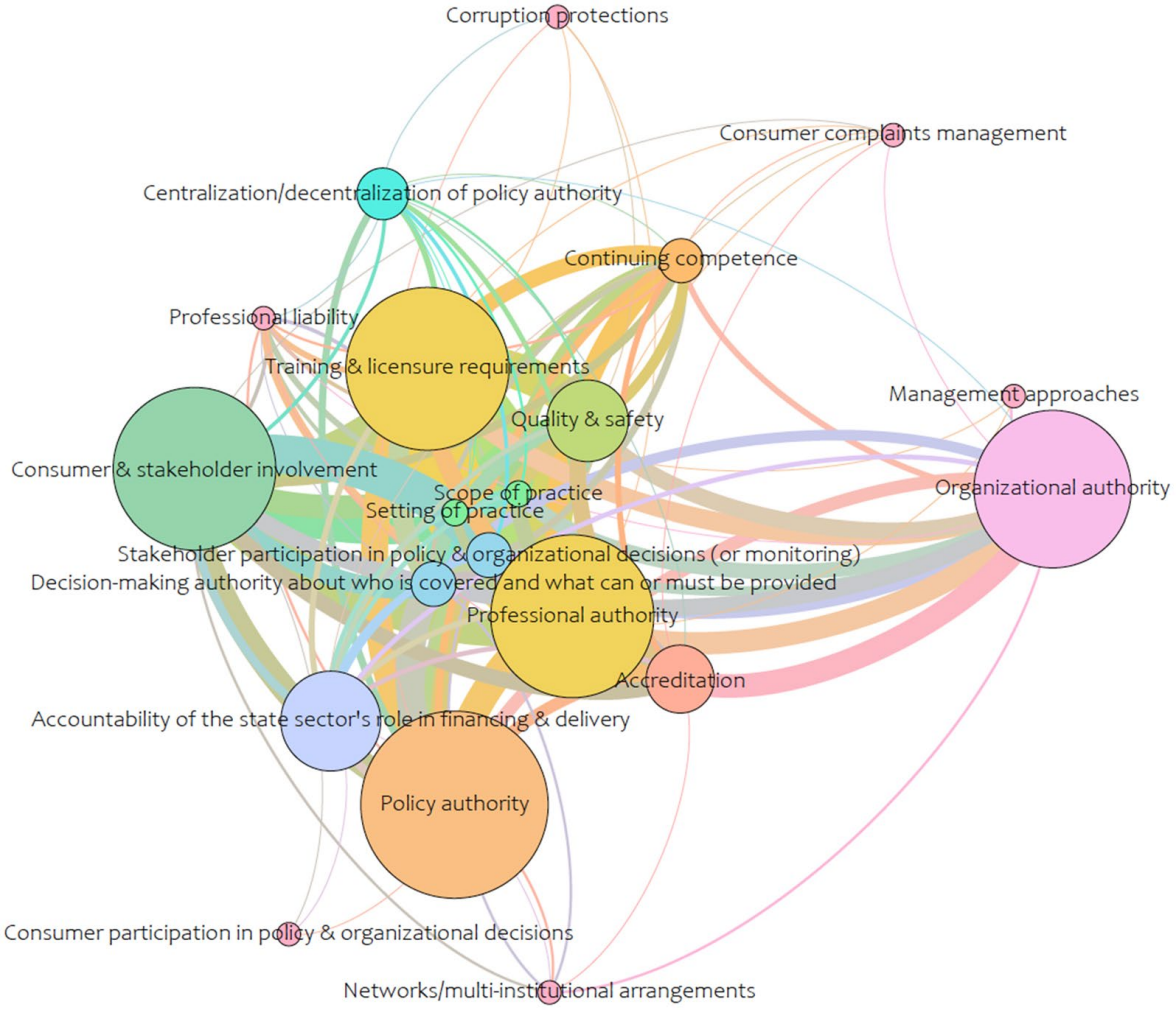
Network analysis of governance arrangements found in the literature on the roles of midwives in health systems

**Fig. 2:**
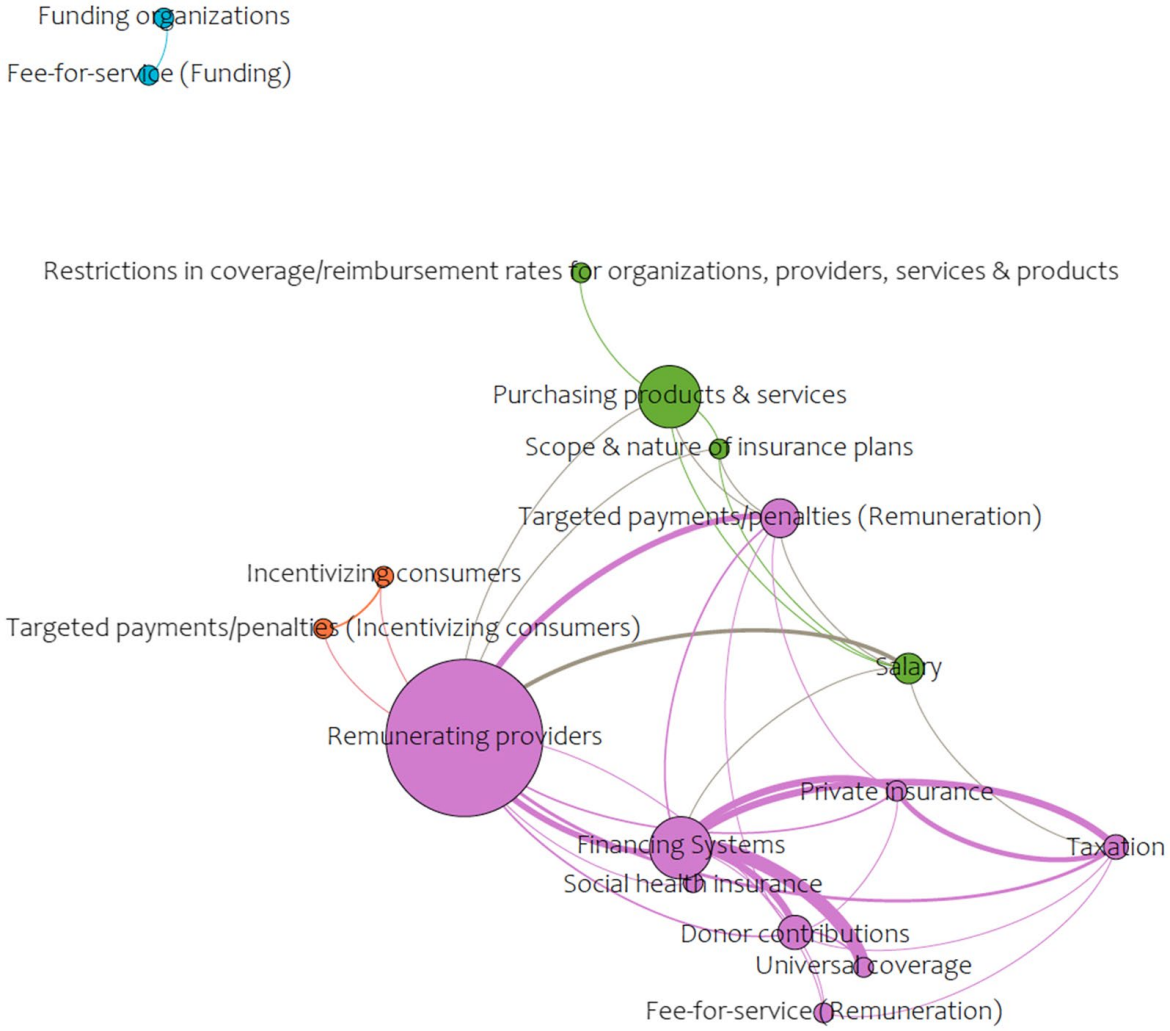
Network analysis of financial arrangements found in the literature on the roles of midwives in health systems

**Fig. 3:**
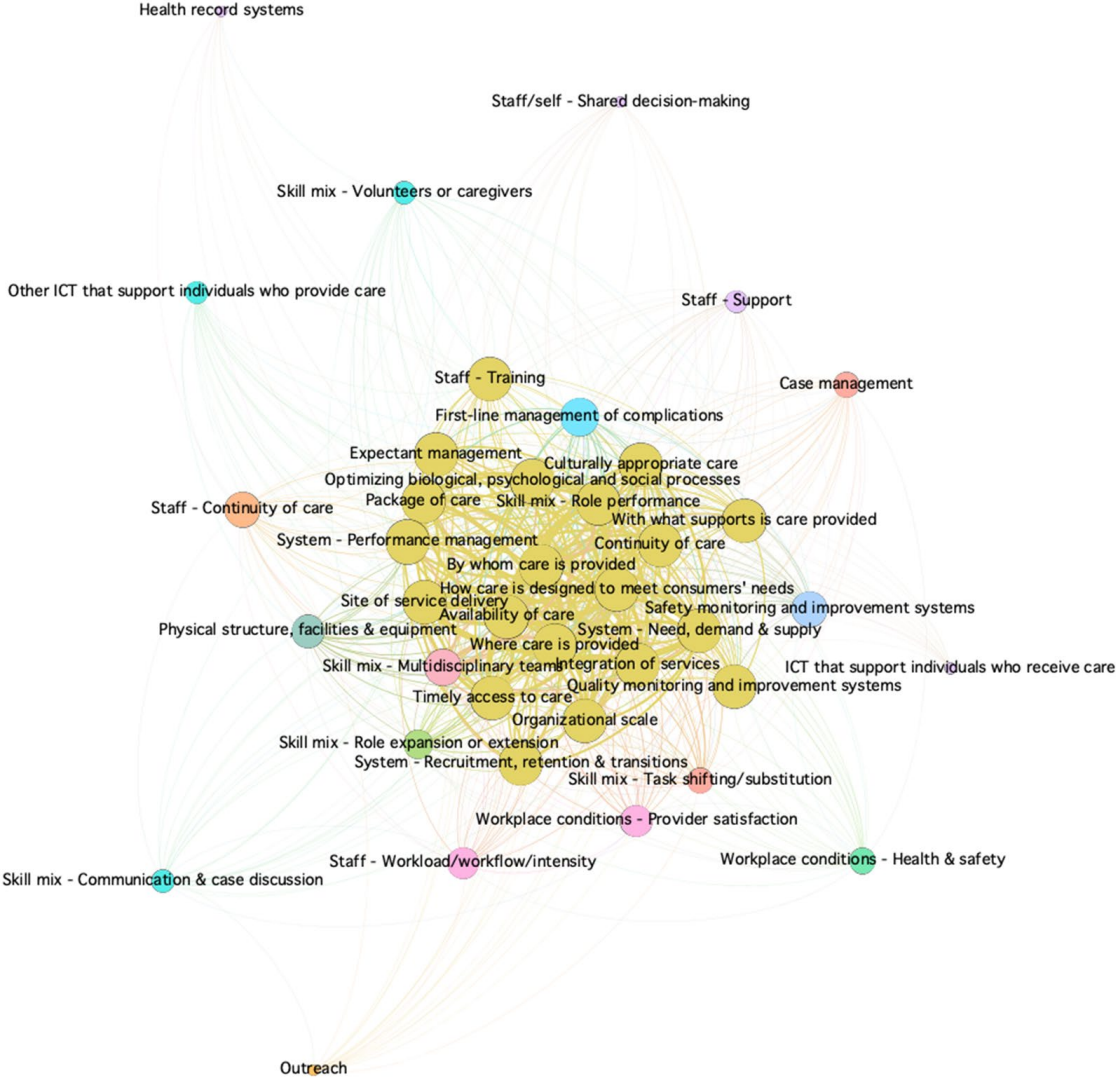
Network analysis of delivery arrangements found in the literature on the roles of midwives in health systems

**Fig. 4:**
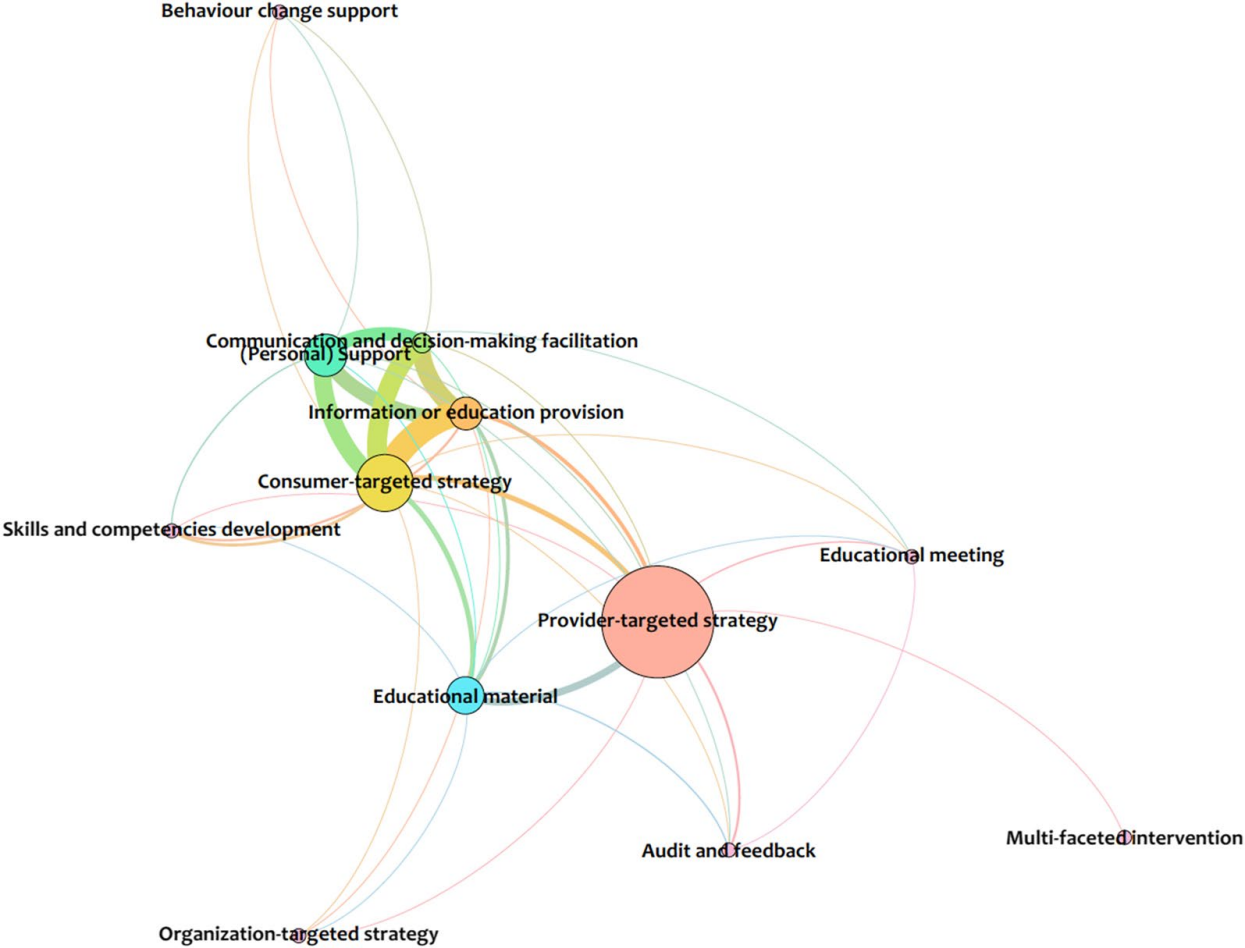
Network analysis of implementation strategies found in the literature on the roles of midwives in health systems

Coding for the network analysis form was informed by two guiding frameworks: (1) components of the framework for quality maternal and newborn care [12]; and (2) ‘health systems arrangements’ taxonomy developed by the McMaster Health Forum that includes governance arrangements, financial arrangements, delivery, and implementation strategy [23]. The taxonomy lends itself to a network analysis because of its level of specificity, which captures the range and breadth of policy levers available to health systems [24]. The analysis represented the relationship between the compass question and the ‘taxonomy. The analysis was separated to examine the arrangements and implementation strategies separately, as combining them in a single analysis yielded too large a network (see Figs. 1–4).

Gephi (version 0.9.3), an open-source free software package and visualization tool, was used for the analysis. Comma-separated values files, for nodes and edges within the areas of the health system arrangements, were created from Excel and imported into Gephi. The Force Atlas layout was used and statistics were run for the network diameter (the maximum number of steps required to cross the network) to calculate betweenness centrality, using an undirected graph. Betweenness centrality appearance functions were used to change the size of the nodes, meaning that the smaller nodes in the figures have a lower betweenness centrality and the bigger nodes are higher. Lastly, the modularity function was run for community detection to better understand what clusters of nodes are grouped together.

### Lessons learned through combining a CIS with a network analysis

Within the literature reviewed, the most explored area was within delivery arrangements (39 nodes, 626 edges), with a more limited understanding of the governance arrangements (20 nodes, 128 edges) involved, and a minimal conceptual understanding of the necessary financial arrangements (17 nodes, 37 edges) and implementation strategies (12 nodes, 38 edges) for the integration of the profession. High betweenness was found in the delivery arrangements network (Fig. 3) for concepts that related to the ways the midwifery model of care is designed to meet clients’ needs (e.g., how care is delivered, availability of services, and context(s) that midwives provide care), as well as midwifery human resources (e.g., need, demand, supply, and recruitment and retention). The network for governance arrangements (Fig. 2) was much smaller and focused on training and licensure requirements related to professional authority (i.e., who makes professional decisions), policy authority (i.e., who makes policy decisions), and organizational authority (i.e., organizational decisions regarding services). The financial arrangements network (Fig. 2) was even smaller and centered on remuneration mechanisms for midwives within the health system. Lastly, implementation strategies, the smallest network (Fig. 4), concentrated on midwifery-targeted implementation strategies (e.g., clinical care recommendations such as clinical practice guidelines).

Identifying where the gaps in understanding lie within the health system is a critical finding for the profession itself and for health system decisionmakers. We learned that there is very little coverage of governance arrangements in the literature, meaning that there is limited knowledge of how policy decisions regarding the profession within the health system are being made (e.g., the level of government that is accountable for decision making regarding midwifery service delivery), as well as how the profession is regulated, and who makes professional decisions regarding midwifery (e.g., scope-of-practice, setting of practice, and continuing competence). Financial arrangements was one of the sparsest networks and many of the concepts related to how the health system is financed, and the specific ways in which midwives are paid within the health system (i.e., salaried, capitation, or fee-for-services models) were missing, which is arguably a crucial component to integration of any health profession.

Understanding the relevant literature using the taxonomy helps to orient midwifery as a profession and supports decision makers (i.e., national ministries of health and United Nations agencies), to work towards global targets (e.g., Sustainable Development Goals). Despite international policy supporting midwifery as an autonomous profession to nursing, midwifery is most often not autonomously regulated nor appropriately integrated, which is confirmed by our findings [25]. Concepts related to legalisation, policies, and procedures which are vital to creating midwifery regulatory frameworks were missing.

### Comparing the value of combining evidence syntheses with network analyses to other approaches

There is a growing field in bibliometrics of combining literature reviews with social network analysis, which have been used to analyse citation records to systematically describe history, patterns of use, research topics, and collaborations among researchers and institutions [26, 27]. Bibliometric approaches are varied but provide relatively quick automated ways to quantitatively analyse publications [28]. A limitation is that bibliometric indicators can be misinterpreted and conclusions drawn that the approach was not intended to measure [28]. Recent uses of computational methods using topic modelling may allow researchers to classify the substantive focus of a large number of research outputs and model relationships within research areas [29]. However, such methods still require domain specific knowledge to interpret classifications and may recover classifications that provide little insight into the review’s primary research questions [30].

Our approach provides an opportunity to carry out a more thorough analysis than that of bibliometric measures. The main advantage, as opposed to fully automated approach, is the investment of time and multiple independent reviewers to code the documents manually based on detailed established taxonomies. Our research objective was to create a theoretical framework in an area where there is limited understanding. We used the areas of expertise of the authors (health systems, health policy, clinical practice, and political science) to inform the selection documents and created theoretical propositions to guide and code the literature. While this approach is more labour intensive, we have increased confidence in our data extraction based on theory, which lends tremendous value and conceptual accuracy. Using a bibliometrics approach would have likely excluded relevant documents, and because we were purposefully drawing from a range of literature, we were able to prioritize the inclusion of empirical articles, which are the types more likely to address health systems.

Our research note outlines the value of combing a CIS with a network analysis. Our findings tell us what health system arrangements have been explored in the literature, how these concepts are related, and identifies important areas that are absent. Future research could use igraph R package to model the networks, which would allow greater options in terms of performance.

The results of the network analysis have real world applications in global SRHR. Midwives contributions are often limited by lack of professional recognition and disempowerment of the profession driven by the intersectionality of gender, sociopolitical, professional, and economic factors [11, 31–34]. Our findings highlight the core contextual factors that governments can use to best leverage the professions’ position in health systems when working to improve SRHR and meet global agendas.

The State of the World’s Midwifery 2021 report highlights that there is a worldwide shortage of midwives and that big investments are needed to meet SRHR needs [10]. The report identifies four core strategic areas of midwifery that are in need of significant supports: (1) health workforce planning, data systems, management, and regulatory systems (governance arrangements); (2) high-quality educators and midwifery education programs (governance arrangements); (3) midwifery-led service delivery including partnerships, interprofessional care, expanded scopes, and pandemic responsiveness (governance, financial, and delivery arrangements); and (4) midwifery leadership across all levels of health systems (governance and financial arrangements) [10]. These strategic investment areas match the conceptual gaps identified in the network analysis. Without the research evidence to inform action, these barriers to the profession meeting its full potential and global SRHR health targets will persist.

## Limitations

We recognize that search strategy may not have fully covered the diverse terminology used to refer to midwifery. However, we consulted with a librarian to ensure that the search strategy was as inclusive as possible.

## Data Availability

The dataset generated during the current study are not publicly available but are available from the corresponding author on reasonable request.

## Abbreviations

CIS: Critical interpretive synthesis
SRHR: Sexual and reproductive health and rights

## References

[1] Booth A, Noyes J, Flemming K, Gerhardus A, Wahlster P, Van Der Wilt GJ, et al. Guidance on choosing qualitative evidence synthesis methods for use in health technology assessments of complex interventions. Bremen (DE): Integrate-HTA. 2016. https://www.researchgate.net/publication/298743768_Guidance_on_choosing_qualitative_evidence_synthesis_methods_for_use_in_health_technology_assessments_of_complex_intervention

[2] Kastner M, Antony J, Soobiah C, Straus SE, Tricco AC (2016). Conceptual recommendations for selecting the most appropriate knowledge synthesis method to answer research questions related to complex evidence. J Clin Epidemiol.

[3] Dixon-Woods M, Bonas S, Booth A, Jones DR, Miller T, Sutton AJ (2006). How can systematic reviews incorporate qualitative research? A critical perspective. Qual Res.

[4] Dixon-Woods M, Cavers D, Agarwal S, Annandale E, Arthur A, Harvey J (2006). Conducting a critical interpretive synthesis of the literature on access to healthcare by vulnerable groups. BMC Med Res Methodol.

[5] Moat KA, Lavis JN, Abelson J (2013). How contexts and issues influence the use of policy-relevant research syntheses: a critical interpretive synthesis. Milbank Q.

[6] Depraetere J, Vandeviver C, Keygnaert I, Beken TV. The critical interpretive synthesis: An assessment of reporting practices. Int J Soc Res Methodol. 2020:1–21. https://www.tandfonline.com/doi/full/10.1080/13645579.2020.1799637

[7] Golbeck J (2013). Analyzing the social web.

[8] Thilagam PS (2010). Applications of social network analysis.

[9] Eck NJV, Waltman L (2014). Visualizing bibliometric networks.

[10] United Nations Population Fund ICoM (2021). World Health Organization, State of the world’s midwifery 2021.

[11] Renfrew MJ, Ateva E, Dennis-Antwi JA, Davis D, Dixon L, Johnson P (2019). Midwifery is a vital solution-what is holding back global progress?. Birth.

[12] Renfrew MJ, McFadden A, Bastos MH, Campbell J, Channon AA, Cheung NF (2014). Midwifery and quality care: findings from a new evidence-informed framework for maternal and newborn care. Lancet.

[13] Starrs AM, Ezeh AC, Barker G, Basu A, Bertrand JT, Blum R (2018). Accelerate progress—sexual and reproductive health and rights for all: report of the guttmacher-lancet commission. Lancet.

[14] ten Hoope-Bender P, de Bernis L, Campbell J, Downe S, Fauveau V, Fogstad H (2014). Improvement of maternal and newborn health through midwifery. Lancet.

[15] UNFPA. The Maternal and Newborn health thematic fund annual report 2019. New York: United Nations population fund; 2019.

[16] Nove A, Friberg IK, de Bernis L, McConville F, Moran AC, Najjemba M (2021). Potential impact of midwives in preventing and reducing maternal and neonatal mortality and stillbirths: a lives saved tool modelling study. Lancet Glob Health.

[17] Sandall J, Soltani H, Gates S, Shennan A, Devane D (2016). Midwife-led continuity models versus other models of care for childbearing women. Cochrane Database Syst Rev.

[18] Mattison CA, Lavis JN, Wilson MG, Hutton EK, Dion ML (2020). A critical interpretive synthesis of the roles of midwives in health systems. Health Research Policy and Systems..

[19] Marin A, Wellman B, Scott J, Carrington PJ (2011). Social network analysis: An introduction. The SAGE handbook of social network analysis.

[20] Scott J, Carrington PJ, Scott J, Carrington PJ (2011). Introduction. The SAGE handbook of social network analysis.

[21] Borgatti SP, Mehra A, Brass DJ, Labianca G (2009). Network analysis in the social sciences. Science.

[22] Cherven K (2015). Mastering gephi network visualization.

[23] Lavis JN. Health systems evidence: Taxonomy of governance, financial and delivery arrangements and implementation strategies within health systems. Hamilton, Canada: McMaster health forum; 2017. https://www.mcmasterforum.org/docs/default-source/resources/16_hse_taxonomy.pdf?sfvrsn=281c55d5_7

[24] Lavis JN, Wilson MG, Moat KA, Hammill AC, Boyko JA, Grimshaw JM (2015). Developing and refining the methods for a ‘one-stop shop’for research evidence about health systems. Health Res Policy and Syst.

[25] Lopes SC, Nove A, ten Hoope-Bender P, de Bernis L, Bokosi M, Moyo NT (2016). A descriptive analysis of midwifery education, regulation and association in 73 countries: the baseline for a post-2015 pathway. Hum Resour Health.

[26] Liu J, Yu F, Song L (2020). A systematic investigation on the research publications that have used the medical expenditure panel survey (MEPS) data through a bibliometrics approach. Library Hi Tech.

[27] Cowhitt T, Butler T, Wilson E (2020). Using social network analysis to complete literature reviews: a new systematic approach for independent researchers to detect and interpret prominent research programs within large collections of relevant literature. Int J Soc Res Methodol.

[28] Belter CW (2015). Bibliometric indicators: opportunities and limits. JMLA.

[29] Thakur K, Kumar V (2021). Application of text mining techniques on scholarly research articles: Methods and tools. New Rev Acad Librariansh.

[30] Chen J, Wei W, Guo C, Tang L, Sun L (2017). Textual analysis and visualization of research trends in data mining for electronic health records. Health Policy Technol.

[31] Day-Stirk F, Kehoe S, Meilson J, Norman J (2010). Capacity development—a midwifery perspective. Maternal and infant deaths: chasing millennium development goals 4 and 5.

[32] Goodman S (2007). Piercing the veil: the marginalization of midwives in the United States. Soc Sci Med.

[33] UNFPA-ICM. Investing in midwives and others with midwifery skills to save the lives of mothers and newborns and improve their health. New York: United Nations population Fund; 2006. https://www.cambridge.org/ca/academic/subjects/medicine/obstetrics-and-gynecology-reproductive-medicine/maternal-and-infant-deaths-chasing-millennium-development-goals-4-and-5?format=PB

[34] Lopes SC, Titulaer P, Bokosi M, Homer CS, ten Hoope-Bender P (2015). The involvement of midwives associations in policy and planning about the midwifery workforce: a global survey. Midwifery.

